# Assessing public perception and awareness of UK mandatory calorie labeling in the out‐of‐home sector: Using Twitter and Google trends data

**DOI:** 10.1002/osp4.674

**Published:** 2023-04-23

**Authors:** Megan Polden, Eric Robinson, Andrew Jones

**Affiliations:** ^1^ Department of Primary Care and Mental Health University of Liverpool Liverpool UK; ^2^ Department of Psychology University of Liverpool Liverpool UK; ^3^ School of Psychology Liverpool John Moores University Liverpool UK

**Keywords:** calorie labeling, Google trends, public health, Twitter

## Abstract

**Objectives:**

In 2021 the UK government announced a new obesity policy requiring large out‐of‐home food outlets to provide mandatory in‐store calorie labeling on food and drink items. Public acceptability and engagement with obesity policies could influence the level of impact on wider public health particularly with population‐level policies such as calorie labeling. This study aimed to examine public responses and awareness of the policy using social media (Twitter) comments and Google trends data.

**Methods:**

This study examined responses to social media posts on Twitter (tweets) from the UK Department of Health and Social Care detailing the policy, implementation date and post‐implementation information about the policy's enforcement. The sentiments of the tweets were coded and the number of likes and replies extracted. This study utilized google trends to examine public awareness of the policy by extracting weekly relative search volume for relevant phrases such as “calorie labeling.”

**Results:**

From the 276 replies/quote‐tweet extracted, the majority expressed a negative sentiment toward the policy (*N* = 197/71.4%). There were fewer tweets expressing a positive sentiment (*N* = 25/8.7%) and a neutral/no sentiment (*N* = 54/19.6%). There was no difference in the number of “likes” or retweets between tweets expressing positive or negative sentiments. Five themes were identified expressing negative sentiments (most common being negative impacts on eating disorders). Google trends data revealed increased searches for “calorie labels/labeling” during the week of the policy enforcement compared to previous weeks in the last 5 years but no significant differences in searches for specific menu calorie labeling.

**Conclusions:**

This analysis revealed negative sentiment toward and increased searching of calorie labeling information during the announcement and implementation of the 2021 mandatory calorie labeling policy in England. A greater understanding of public responses to calorie labeling policies may help tailor future policies and public communication strategies.

## Graphical abstract

In 2021 the UK government announced a new obesity policy requiring large out‐of‐home food outlets to provide mandatory in‐store calorie labeling on food and drink items. This study examines public responses and awareness of the policy pre and post‐implementation using Twitter comments and Google trends data.

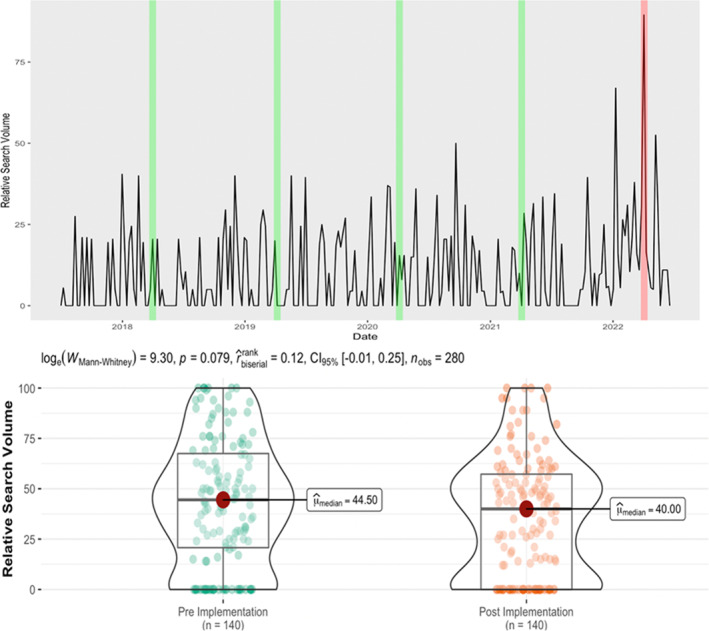

## 1 INTRODUCTION

Out of Home Food Sector (OHFS) refers to food that is sold in the out of the home sector for immediate consumption. The wider food environment, particularly the OHFS, is thought to be a key contributing factor to increased prevalence of overweight and obesity both globally,^1^ and in the UK.^2^ This is because food products and meals sold in the OHFS tends to contain higher energy content than food prepared at home^3^ and are often over the UK recommendation of 600 calories for a lunch or evening meal.^4^, ^5^ The availability of these highly calorific dishes is problematic due to the frequency that people consume food out‐of‐home,^6^, ^7^ with more frequent consumption associated with an increased risk of developing overweight or obesity.^8^

In 2011 the UK public health responsibility deal was established by the UK government, in collaboration with members of the food industry, to address public health and combat rising obesity. A key component of this deal was for businesses to voluntarily provide calorie information on menus for food and non‐alcoholic drinks. Whilst several restaurants and cafes signed the calorie labeling pledge, a 2018 assessment found that less than 20% of UK restaurants provided any in‐store calorie content information.^9^ Criticisms of the deal included allowing the industry to “*appear to be helping improve public health without having to do very much*.”^10^ Therefore, in 2021, the UK government announced a *mandatory* calorie labeling policy requiring large‐scale food businesses (>250 employees) in England, to provide calorie labeling on food and non‐alcoholic drink items to address public health and combat rising obesity with the policy coming into effect in April 2022. The policy requires outlets in England selling food for immediate consumption (cafes, fast‐food outlets, sit‐down restaurants, pubs) to provide calorie labeling on all unpackaged food and non‐alcoholic drink items on the menu for more than 30 days per year, alongside contextual information on recommended calorie consumption. Guidelines state that labeling should be clearly presented, legible and provided for all eligible food and drink items.

Similar policies have recently been passed in the USA,^11^ Australia^12^ and parts of Canada.^13^ Calorie labeling can reduce calorie consumption in two ways; by influencing individuals' choices^11^ and through menu reformulation.^7^, ^14^, ^15^ One systematic review found that calorie labeling on menus influenced consumer purchasing and resulted in a 7.8% reduction in the number of calories purchased.^16^ Furthermore, studies examining the nutritional content of menu items sold found that restaurants with calorie labeling had 45% less fat and 60% less salt^15^ compared to restaurants without calorie labeling. Other studies have shown a reduction in calorie content following the implementation of calorie labels on prepared supermarket foods and concluded that such menu reformations could lead to reductions in overall calorie intake.^14^ These studies demonstrate how calorie labeling could have impacts on wider public health through the combination of menu reformulation and influencing individuals' choices. However, it should be noted that many studies have showed no significant impact on food ordering.^17^, ^18^, ^19^, ^20^, ^21^ The true extent of mandatory calorie labeling on long‐term public health is currently unknown and it is unclear whether mandatory calorie labeling will have a substantial and prolonged public health benefits as a standalone policy.^22^

Positive public perceptions, awareness and engagement with health policies, particularly ones such as calorie labeling that rely on consumers noticing and use of provided information, may prove vital for a wider impact on public health and obesity levels.^23^ Research from Saudi Arabia found that 85% of people surveyed thought food labeling in restaurants was useful, however, only 43% stated that they always or usually use food labeling when making their purchases.^24^ It was identified that the main barrier for not using food labeling was time constraints (25%) followed by difficulty to use (24%).^24^ Understanding public perceptions and the barriers that prevent consumers from engaging with calorie labeling could help tailor campaigns and educational programs promoting the use of calorie labeling.

Some researchers have suggested that calorie labeling will be ineffective due to a lack of awareness or the noticing of the nutritional information by consumers.^25^, ^26^ Indeed, several studies have demonstrated that when calorie labels are present on menus reported awareness is low,^27^, ^28^, ^29^ however this can be increased following effective social marketing campaigns aiming to raise awareness.^30^ This demonstrates that raising public awareness increases engagement with calorie labeling practices and may increase the potential impact of the policy.

It is also possible that negative attitudes by the general public toward the policy will act as a barrier to long‐term success or continued implementation.^31^ Again, the available evidence is mixed. Beeken and Wardle^32^ demonstrated that 66% of a representative UK sample of 1986 participants, agree/strongly agree with the comment “*The Government should insist that restaurants and takeaways give information on the fat and calorie content of foods*,” which supports findings from other countries (e.g., Canada^33^). However, a recent systematic review suggested one of the main barriers to policy implementation was a lack of customer demand for/interest in menu labeling.^34^ Consumers who do not support the policy or are not aware of it may be less inclined to use calorie information, therefore limiting the impact and reach of the policy.

A major limitation of the current evidence base is that most studies examining public perceptions and awareness of menu labeling have asked the public directly, potentially bringing attention to calorie labeling and increasing desirable responding. For instance, Hobin et al.^35^ demonstrated that in a randomized control trial with a no‐calorie information control group 8% of individuals reported noticing calorie information and 16% perceived the information to influence their purchasing behavior in this group.

Studies that use these methods could result in socially desirable responses and self‐selection of participants into studies.^36^, ^37^ One way to reduce these biases is to unobtrusively examine individuals' attitudes through online discourse via social media and information searching.^38^, ^39^ Twitter is one of the largest social media platforms, and the use of Twitter by both the private and public sectors has rapidly expanded since its inception. Reviews have suggested that local health departments readily use Twitter for one‐way communication of public health‐related topics and organization‐related information,^40^ and it plays a significant role in the dissemination of health information.^41^ It allows for event‐based surveillance,^42^ such as the announcement of policy changes.^43^ For example, Stautz^43^ examined twitter responses to updated alcohol guidelines in the UK, concluding that Twitter comments offer a valuable data source for monitoring public responses to health policy announcements. Similarly, Google is the world's largest search engine and provides data on the frequency of searches made through the “Google trends” tool (trends.google.com). Google trends allows researchers to measure information seeking and consumer search behavior^44^ and this data source has been used to study responses to a range of national public health policies. For example, Tieks et al.^45^ used Google trends data to examine smoking‐related outcomes following a national smoking cessation program (Stoptober) in England and the Netherlands.

In the present research, online data sources from Twitter and Google were used to examine online public sentiment and awareness (i.e., searching for policy related information) of the 2021 national calorie labeling policy implemented in England. Twitter replies to three policy announcement tweets made by the government department responsible for its introduction (Department of Health and Social Care: DHSC), concerning mandatory calorie labeling policy were both quantitatively and qualitatively analyzed. Moreover, Google trends data in the weeks following the announcements were examined to explore potential changes in (i) public awareness of the policy and (ii) interest in the calorie content of menus in food outlets affected by the policy.

## 2 METHODS

### 2.1 Data sources

#### 2.1.1 Twitter

On two occasions prior to the policy implementation (May 2021), the UK DHSC sent tweets detailing the policy and when it would be implemented. Post‐implementation (April 2022) a further tweet was sent stating the policy was now enforced. The UK DHSC has a large (>700,000 followers as of April 2022), active (tweeting multiple times per day), and publicly accessible Twitter account.

In line with previous research^43^ all replies and quote‐tweets were manually extracted for the three tweets bringing the policy to the publics attention. The number of “likes” and “replies” the individual tweets had were also extracted. Data on the pre‐implementation tweets were extracted on 1 April 2022 and the post‐implementation tweets were extracted on 14 April 2022. In total, 276 replies/quote tweets were extracted. Visual examination of the Twitter accounts suggested all users were genuine (e.g., had previously posted on their Twitter account or engaged with others, not solely responded to the Tweets by the DHSC, and did not have a randomly generated user name) and the replies did not follow typical “bot” algorithms, including mass tweeting URLs, uploading of images, or having a large number of similar tweets.^46^

#### 2.1.2 Google trends

Google Trends is a publicly accessible online tool provided by Google Inc (https://trends.google.com/), allowing users to examine the relative search frequency of daily Google search terms. This can be tailored to a specific location (e.g., England) as well as time periods, and which Google product is used to search (e.g., “web search,” “news,” “youtube”). Search results are normalized to represent a 0–100 scale, whereby each search term examined is divided by the total searches in that topic area during the specified time range; otherwise known as the relative search volume (RSV). Searches for a given topic have been used as a proxy for increased public awareness, for example, increased public awareness of Tuberculosis following World Tuberculosis Day.^47^

To examine public awareness of the policy weekly RSV values for the terms “calorie labeling,” “calorie labels” were first extracted from Google trends over a 5‐year period (week commencing 2 July 2017 until the week commencing 18 June 2022). During these time periods we also searched “kcal labeling” and “kcal labels” however there was no search information for these. The RSV were then averaged across both search terms before *Z* scoring the averaged RSV values and examining the data period in which calorie labeling was enforced (3 April 2022 to 9 April 2022). This time period was also descriptively compared to equivalent time periods of previous years.

To examine public interest in the calorie content of menus subjected to the policy the search terms were extracted from Google trends for 10 businesses with >250 employees that would be subject to the policy, followed by the word “calories.” It was reasoned this was a good proxy for awareness of the policy, as on the days following implementation the policy was covered in a number of large media sources including The Guardian (“*From today, large UK restaurants and cafes have to display calorie counts on their menus as part of the government's drive to tackle obesity”—* https://www.theguardian.com/society/2022/apr/06/calorific‐which‐high‐street‐meals‐are‐the‐most‐and‐least‐fattening), *BBC news* (*“Calories now appear on menus of large restaurant chains”* https://www.bbc.co.uk/news/business‐60989825) *and Daily Mail* (*“Restaurant Chains Print Calorie Counts on Menus from TODAY*” https://www.dailymail.co.uk/news/article‐10691235/Restaurant‐chains‐print‐calorie‐counts‐menus‐TODAY‐industry‐leader‐say‐WONT‐tackle‐obesity.html). The study focused on popular, well‐known businesses (known by only one name, e.g., not a pub chain with premises with individual names such as Weatherspoons or Hungry Horse) to ensure individuals would likely be searching for information on these businesses. A mix of typical fast food and counter service restaurants were chosen, with different types of food served. Our searches included: McDonald's, KFC, Burger King, Nandos, Pizza Hut, Leon, Bella Italia, Starbucks, and Subway. Our search strategy was limited to two weeks prior to the policy implementation date (23 March 2022 to 05 April 2022) and two weeks following the implementation (06 April 2022 to 19 April 2022).

### 2.2 Analytic procedure

#### 2.2.1 Twitter

For each tweet, or quote tweet the sentiment of the tweet was coded using the same guidelines as Stautz et al.^43^ Tweets were manually coded as positive, negative or neutral/no‐sentiment, by two authors independently (AJ and MP). Positive tweets had to communicate a positive appraisal of the policy or the proposed effects (e.g., “*This is a great idea and will really help people to make healthy choices*”), whereas negative tweets had to communicate a negative appraisal of the policy or the proposed effects (e.g., “*This is an awful idea and will have a negative impact on peoples food choices*”). Tweets that were neutral in sentiment or did not have clear sentiment were coded as such. There was a high degree of agreement between the two coders (>90%). All disagreements were resolved between AJ and MP. Differences in the number of likes and retweets between tweets expressing positive and negative sentiment using Mann‐Whitney tests were also examined.

Following sentiment analyses, Thematic Analysis^48^ was conducted on the tweets separately for those displaying positive and negative sentiment. Thematic analysis is the process of identifying themes or patterns within qualitative data and has a high degree of flexibility across different types of data.^49^ Braun and Clarke's^49^ steps were followed for the analyses, which include; becoming familiar with the data, generating initial codes; searching for themes; reviewing the themes; defining the themes, and dissemination of themes. Thematic analysis was conducted by AJ and MP in isolation. Disagreements were discussed and resolved before agreement on the final themes.

In line with guidelines by Rivers and Lewis,^50^ direct quotes or screen names are not provided to aid in the concealment of the original tweeters. Furthermore, any tweets which were protected (e.g., tweeted through a private account) were not accessed. To aid interpretation the content of the original tweets have been slightly amended to preserve anonymity but to maintain both sentiment and theme. This ensures that information cannot be entered into a search engine to trace back to original tweeters.

#### 2.2.2 Google trends

Using the “gtrendR” R package^51^ the weekly RSVs for “calorie labeling” and “calorie labels” were extracted. The weekly data was Z‐scored before generating the *p*‐value from the *Z* score of week of interest. The RSVs for “[restaurant name] calories” were then extracted during the search period 23 March 2022 to 19 April 2022. This provided us with 10 restaurants × 28 days = 280 RSV data points. To compare search volumes, the 2 weeks prior to the policy and post‐policy implementation were split and the search volumes compared using the Mann‐Whitney test. The RSVs were also combined for each out‐of‐home establishment on a daily basis (Averaging RSV across the 10 restaurants) and correlated this time (day 1–28) using Spearman's correlation. Data and analysis code is available on the Open Science Framework (https://osf.io/bu5jw/).

## 3 RESULTS

### 3.1 Twitter sentiment analysis

The original DHSC tweets received a total of 186 likes, as of June 2022. From the 276 replies/quote‐tweets the majority expressed a negative sentiment (*N* = 197/71.4%). A much smaller number expressed a positive sentiment (*N* = 25/8.7%), and neutral/no‐sentiment tweets (*N* = 54/19.6%). There was no difference in the number of “likes” for tweets expressing negative (min = 0, max = 87, median = 1, mean = 4.06) versus positive sentiment (min = 0, max = 41, median = 1, mean = 3.65: *W* = 2069.5, *p* = 0.475). There was also no difference in the number of retweets of tweets expressing a negative sentiment (min = 0, max = 40, median = 0, mean = 0.54) versus positive sentiment (min = 0, max = 6, median = 0, mean = 0.56: *W* = 2400, *p* = 0.509)

#### 3.1.1 Themes

Five themes were identified in tweets expressing negative sentiment. These were “Impact on Eating Disorders,” “Ignoring Experts,” “Nanny state,” “Calorie counting,” “Ineffective/Doesn't go far enough/Will be ignored” (see Table 1). Due to a much smaller number of tweets, there were no clear themes of positive sentiment.

**TABLE 1:** Description and example tweets for identified themes.

Negative tweets			
Theme	Description	Example tweets	Number of tweets
Impact on eating disorders	The policy will negatively impact those with eating disorders or increase the prevalence of eating disorders	“*This will cause harm. People won't make ‘healthy choices’ it will lead to anxiety and guilt, which will increase eating disorders*” “*This will be awful for so many people who have anxiety around food*”	65
Calorie counting	The policy will lead to individual's calorie counting, which is not an effective weight‐loss tool	“*Not all calories are the same. 200 calories from a doughnut is much worse than 200 calories from fish. Quality is what is important*” “*The focus should be on nutritional values, not calorie intake if we want to reduce the burden on the NHS*”	10
It will be ignored/ineffective	The policy will not be effective, either because it doesn't go far enough or because individuals won't use the calorie information	“*Why only big chains? I only eat at independent restaurants, so I will be missing this information*” “*Calorie labels will not put me off eating unhealthy food. I eat unhealthy food because it is delicious and I want to*”	32
Nanny state	The policy is interfering with private businesses and impacting individual's personal choices	“*This creates even more legislation for restaurants and the hospitality sector. People should use their own judgement to eat healthily and exercise*” “*Please just stop interfering with our lives and let us make our own decisions*”	16
Ignoring experts	The policy has been implemented, despite expert advice/recommendations otherwise	“*This is going ahead, despite experts and campaigners recommending otherwise!*” “*Eating disorder specialists should have been listened to*”	9

### 3.2 Google trends in searches for out‐of‐home calories

**FIGURE 1:**
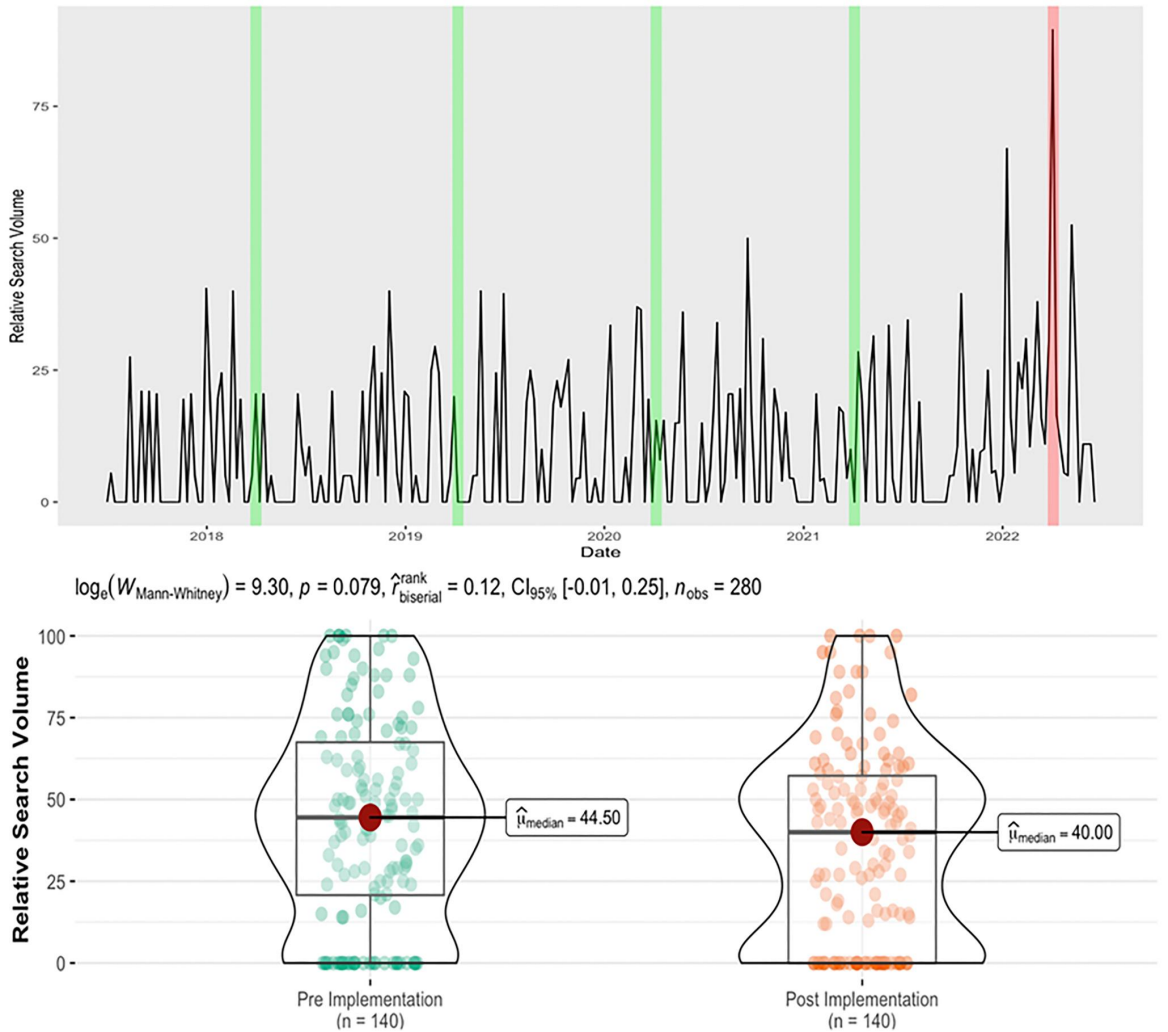
RSV for out‐of‐home outlet calories for the 2 weeks post‐implementation versus pre‐implementation (top panel) and RSV across the 2‐week search period (bottom panel). Top panel = Relative Search volumes of Calorie Labeling/Labels over the previous 5‐years. The red vertical line represents the period of policy enactment, green lines represent the same time period in previous years. Bottom panel = Relative Search Volumes of 10 popular out‐of‐home eating outlets and “calories” in Google Trends, by pre‐policy and post‐policy implementation. RSV, relative search volume.

Searches for 'calorie labels/labeling' were significantly greater during the week of the policy enforcement (RSV = 89.5, *Z* Score = 5.92, *p* < 0.001: See Figure 1 top panel), and higher than any other week during the previous 5 years. In 2018 during the same time period the average RSV = 20.5, in 2019 = 20.0, in 2020 = 15.5 and in 2021 = 0. This suggests individuals included in this assessment demonstrated some awareness of the wider policy enactment. However, there was no significant difference in searches for specific menu calorie labeling in the weeks leading up to and post‐intervention (Figure 1: bottom panel).

## 4 DISCUSSION

This study examined selected public responses to the 2022 mandatory calorie labeling policy in England by using openly available data collected from Twitter and Google trends. Two hundred and seventy‐six Twitter replies were analyzed from three Twitter posts made by the DHSC about the calorie labeling policy. Most of the tweets expressed negative sentiments toward mandatory calorie labeling in England (71.4%). Five themes were identified expressing negative sentiment toward the policy, including: “Impact on eating disorders,” “Ignoring experts,” “Nanny state,” “Calorie counting,” and “Ineffective/Doesn't go far enough/Will be ignored.”

Negative sentiment was related to the potential adverse effect of calorie labeling on individuals with eating disorders, with concerns that calorie labeling will increase the prevalence of eating disorders, impact recovery, and increase the likelihood of relapse. UK eating disorder charities have expressed concerns about the policies effect on people with eating disorders and this sentiment is largely reflected in the Twitter responses. “Beat,” the UK eating disorder charity argues against the implementation of mandatory calorie labeling after warning that calorie labeling “risks exacerbating all eating disorders.”^52^ To mitigate the impact on individuals with eating disorders, government guidance encourages businesses to provide menus without calorie labeling on request, however, this is currently not mandatory.^53^ Whilst there is limited evidence of harm from countries that have previously implemented mandatory calorie labeling,^7^ the long‐term impacts of the UK policy on people living with eating disorders needs to be investigated.

In relation to this, a second theme identified was that the policy will be “ineffective/doesn't go far enough/will be ignored.” Multiple tweets expressed negative sentiments reflecting the potential harm toward individuals with eating disorders combined with the view that the policy will be ineffective at achieving aims to reduce obesity levels. This is in line with qualitative evidence in which individuals reported support for calorie labeling policies, but scepticism regarding their effectiveness.^23^ These themes indicate a narrative on social media that the policy will cause considerable adverse effects in individuals with eating disorders, with minimal evidence of benefit to the wider population. Multiple tweets expressed that the policy should include independent businesses to have a wider reach and that limiting the policy to only large businesses is ineffective.

Similarly, the theme “*calorie counting*” included tweets that expressed concerns that the policy approach may be oversimplistic as “*calories in, calories out*” is not a useful explanation of obesity.^25^ Finally, individuals also reported that mandating calorie information impacts businesses' and individuals' free choice (“nanny state politics”). This echoes concerns by businesses relating to the cost and time implications of implementing labeling.^54^ However, on the individual level calorie labeling is not designed to influence free choice, but rather promote a more informed choice.^55^

In summary, most tweets expressed negative sentiments discussing calorie labeling policy with the overarching theme that the policy will be inefficient in reducing obesity levels. However, a minority of tweets (8.7%) were positive toward calorie labeling and there was no difference in how frequently negative versus positive tweets were endorsed by others online. Because polarized opinions (i.e., dislike of calorie labeling) are more likely to be shared on social media, the tendency for Twitter responses to the policy to be largely negative is to be largely expected. Research examining the spread of information on Twitter found that increased negativity (but not positivity) predicted content sharing on Twitter when examining political content.^56^ This study highlights the tendency for negative content to spread further compared to positive content and the nature of public engagement on Twitter. Furthermore, the data is subject to people's tendencies to voice negative opinions than those who hold neutral or positive opinions.^57^, ^58^ Due to this and the self‐selecting nature of Twitter comments included in this study, the present results unlikely provide an accurate and all‐encompassing account of public opinions toward calorie labeling. However, the themes identified in the present study provide valuable insight as addressing concerns may improve the effectiveness of the policy or inform further implementation of calorie labeling policies nationally or internationally.

Data from Google trends indicated that there was an overall increase in searches for calorie labeling around the period of policy enactment, specifically in relation to similar time periods in previous years (likely due to media coverage). However, the policy announcement did not significantly increase interest in calorie content of menu items in food outlets affected by the policy. This alongside research that has found low levels of noticing of calorie information in restaurants may indicate a lack of public interest in calorie content of labeled menu items.^25^, ^29^ A benefit of the measure utilized in this study is the lack of susceptibility to socially desirable reporting, evident in previous studies.^35^ Findings indicate an overall awareness of the policy demonstrated by increased Google searches for “calorie labeling,” however, this did not appear to translate into a wider search interest of calories within the specific outlets impacted by the policy. An increased interest in the energy content of foods in the OHFS has been proposed as a mechanism by which calorie labeling could reduce obesity.^22^ Studies have demonstrated increased noticing rates after social media marketing campaigns^29^ and these may be required to increase engagement and awareness of calorie labeling in the UK.

To our knowledge, this study is the first to examine public responses to the implementation of the 2022 calorie labeling policy in England. The data utilized offers a novel insight into online public responses without requiring participants to self‐select into a study (selection bias) or answer questions in a socially desirable way. However, it should be noted that tweets themselves are limited in length, and often contain unconventionally written expressions that can make it difficult to portray an in‐depth opinion or sentiment.^59^

Furthermore, a limitation is the inability to determine the demographics of the sample and therefore conclusions cannot be drawn on the representativeness or generalizability. The sample utilized was comprised of a small proportion of Twitter and internet users selected for inclusion based on engagement with the identified tweets (and presumably directly following or following others who follow the UK DHSC). Research suggests that men and people from densely populated areas are over‐represented on Twitter and user ethnicities proportions are not representative of the general population.^56^ Mellon and Prosser^60^ suggest that UK Twitter users are younger and have greater education qualifications than the general population.^61^ Although, 92% of adults in the UK reported recent internet use in 2020^62^ and Twitter and Google are among the most popular platforms for searching and opinion sharing in the UK.

Additionally, Twitter replies may provide a useful insight into immediate reactions to presented information, however they do not provide information on the deliberation time before the response or if the individual's opinion altered after more in‐depth deliberation. Evidence suggests that Twitter tends to have more negative communications,^63^ but it is possible that after viewing an announcement tweet and having an immediate negative response, people may seek out further information leading to a changed response, not reflected in the current research. Finally, the Google Trends data does not tell us whether the policy resulted in an increased interest in the calorie content of foods when visiting outlets (or by going straight to the business's website via a direct URL). These instances are not reflected in our findings and therefore a limitation of the present research. Additionally, a minority of food outlets had implemented calorie labeling ahead of the policy implementation, so it is possible people were already aware of calorie content in these restaurants prior to the announcements.

Twitter replies and Google trends data can offer valuable insight into public interest and opinions on health policies. In the present study, several negative themes were identified relating to the announcement and implementation of the 2021 mandatory calorie labeling policy in England when examining social media data. The study also found that although internet searches for calorie labeling increased upon implementation, there was no evidence suggesting that searches for calorie content information of OHFS foods increased. Future research is required to investigate whether the policy leads to a longer‐term increase in public interest of OHFS food calorie content. Furthermore, research should examine whether public perceptions of calorie labeling affects the level of reach and impact of the policy on public health.

## AUTHOR CONTRIBUTIONS

Conceptualization and methodology design contributions and project administration contributions were made by Megan Polden, Andrew Jones and Eric Robinson. The formal analysis, data curation, and original draft preparation contributions were made by Megan Polden and Andrew Jones. Supervision and funding acquisition were conducted by Andrew Jones and Eric Robinson. All authors contributed to revising the manuscript. All authors read and agreed to the published version of the manuscript.

## CONFLICT OF INTEREST STATEMENT

The authors declare no conflict of interest.
